# 

**Published:** 1914-01-10

**Authors:** 


### CONTENTS.

| | PAGE |
|---|---|
| **Effects of Fog in Institutions** ... ... ... | 383 |
| **The New Poor-Law Orders** ... ... ... | 384 |
| **Hospital and Institutional News** ... ... ... | 385 |

The New Year Honours—A Hospital Knight—Honour for Medical Missions—Medical Witnesses in Coroners' Courts—New Mental Medical Officers' Association—Lock Hospitals left in the Lurch—Night Clinics and the London Lock Hospital—Post-Graduation Lecture on Children's Diseases—Ambulance Hospitals in South Wales—Panel Practice and Poor Law Infirmaries—A Panel Doctor's Circular—Honour for Hospital Founder—Routine and Research at Glasgow—Death of the Inventor of "Rest-cure"—Increase of Leprosy in France—Waste Products as a Source of Income—A Query on Christmas Rejoicings—The Staff Ladder at Swansea Hospital—Salaries and the Saturday Fund—The Hospital Cook—Cottage Hospitals and Medical Staffs—X-Ray Cancers and Radiation Therapy—The Workpeople's Fund at Leicester—A Curious Source of Income—This Week's Drug Market.

| | PAGE |
|---|---|
| **Grave Position at St. George's Hospital** ... | 391 |

The Causes of the Present Deadlock.
Cease Personal Intrigues.
Make the Hospital's Interests Paramount.

[*See over.*]

### CONTENTS—(*continued*).

| | PAGE |
|---|---|
| **The Editor's Letter-Box** ... ... ... ... ... | 393 |
| Patients and their Beauty Sleep. | |
| Insured Patients. | |
| **State Hospitals** ... ... ... ... ... | 394 |
| Why English Men and Women Can Never Suffer Them. | |
| **Ionic Treatment of Dental Sepsis** ... ... | 395 |
| The Prevalence and Prevention of the Disease | |
| **The Evolution of the Isolation Hospital** ... | 396 |
| By the late H. FRANKLIN PARSONS, M.D., D.P.H. | |
| **Report on West Norfolk and Lynn Hospital, King's Lynn** ... ... ... ... ... | 397 |
| By Sir HENRY BURDETT, K.C.B., K.C.V.O. | |
| **Appointments** ... ... ... ... ... ... | 398 |
| **National Insurance Act Developments** ... ... | 399 |
| The Investment of Funds. | |
| **The London Ambulance Question** ... ... | 401 |
| Its History and Present Position. | |
| **British Workers in Foreign Countries** ... ... | 402 |
| Hospital Life in Kashmir. | |
| **The Institutional Library** ... ... ... ... | 403 |
| The Sanatorium Routine. | |
| A Handbook of Practical Points. | |
| A Manual of Medical Treatment. | |
| The Nauheim Treatment. | |
| The Way of Success. | |
| **The New Poor-Law Orders** ... ... ... | 405 |
| Their Substance and Effect on Infirmary Work. | |
| **Personalia** ... ... ... ... ... ... ... | 406 |
| **The Profession of Medicine** ... ... ... | 407 |
| The National Medical Union. | |
| Panel Work in Theory and Practice. | |
| **The Institutional Worker** ... ... (Suppl.) | 1 |
| Selected Answer to our December Question: Provision against Fire. | |
| **Our Bureau of Information** ... ... ,, | 2 |
| Metal Filament Lamps. | |
| Corridor Decorations. | |
| Employment and Training. | |
| The Sick and in Need. | |
| **Buildings Contemplated and in Progress** ,, | 3 |

